# A meta-analysis of treatment for early-stage cervical cancer: open versus minimally invasive radical trachelectomy

**DOI:** 10.1186/s12884-023-06036-z

**Published:** 2023-10-14

**Authors:** Zi Lv, Yu-ying Wang, Yu-wen Wang, Jun-jie He, Wen-wei Lan, Jia-ying Peng, Zi-han Lin, Ruo-fei Zhu, Jie Zhou, Zi-qi Chen, Ying-hui Jiang, Yi Yuan, Jian Xiong

**Affiliations:** 1 Department of Obstetrics, Guangzhou Women and Children’s Medical Center, Guangzhou Medical University, Guangzhou, China; 2 Department of Obstetrics, Guangzhou Women and Children’s Medical Center Affiliated to Guangzhou Medical University, Guangzhou, China; 3 School of Pediatrics, Guangzhou Medical University, Guangzhou, China; 4 Department of Gynecology, Guangzhou Women and Children’s Medical Center, Guangzhou Medical University, Guangzhou, China; 5 Department of Gynecology, Guangzhou Women and Children’s Medical Center Affiliated to Guangzhou Medical University, Guangzhou, China

**Keywords:** Radical trachelectomy, Cervical cancer, Fertility-sparing surgery, Meta-analysis

## Abstract

**Background:**

In previous systematic reviews, meta-analysis was lacking, resulting in the statistical difference between the data of different surgeries being impossible to judge. This meta-analysis aims to contrast the fertility results and cancer outcomes between open and minimally invasive surgery.

**Method:**

We systematically searched databases including PubMed, Embase, Cochrane, and Scopus to collect studies that included open and minimally invasive radical trachelectomy. A random-effect model calculated the weighted average difference of each primary outcome via Review Manager V.5.4.

**Result:**

Eight studies (1369 patients) were incorporated into our study. For fertility results, the Open group excels MIS group in pregnancies-Third trimester delivery [OR = 2.68; 95% CI (1.29, 5.59); *P* = 0.008]. Nevertheless, there is no statistical difference in clinical pregnancy, miscarriage, and second-trimester rate. Concerning cancer outcomes, no difference was detected in the overall survival [OR = 1.56; 95% CI (0.70, 3.45); *P* = 0.27] and recurrence [OR = 0.63; 95% CI (0.35, 1.12); *P* = 0.12]. Concerning surgery-related outcomes, the comprehensive effects revealed that the estimated blood loss of the Open group was higher than that of the MIS group[MD = 139.40; 95% CI (79.05, 199.75); *P* < 0.0001]. However, there was no difference between the postoperative complication rate in the two groups [OR = 1.52; 95% CI (0.89, 2.60); *P* = 0.12].

**Conclusion:**

This meta-analysis suggested that the fertility result of the Open group may be better than the MIS group, while the MIS group has better surgery-related outcomes. Owing to the poor cases of our study, a more robust conclusion requires more relevant articles in the future.

**Systematic review registration:**

PROSPERO CRD42022352999.

**Supplementary Information:**

The online version contains supplementary material available at 10.1186/s12884-023-06036-z.

## Introduction

Cervical cancer ranked fourth most frequently diagnosed around the world with morbidity of 0.05% in women aged 20 to 45 years old, which is also the second largest cancer in incidence and mortality in regions of lower gross domestic product [1]. Meanwhile, approximately 98.9 thousand women are newly diagnosed with cervical cancer annually in developing countries like China, with 29.7 thousand under the age of 45 [2]. Of note, with the average age of onset dropped by 5–10 years compared to the past ten years in cervical cancer, [3] radical trachelectomy has gradually become a practical option for patients with early-stage cervical cancer (2 cm or less in size, stage IA2 to IB cervical cancers) who wish to preserve fertility [4].

Radical trachelectomy (RT) is one of the most frequently used fertility-sparing surgeries. At present, various surgical modalities of RT, including trans-abdominal (ART), laparoscopic(LRT), and transvaginal (VRT), are conducted around the world. Compared with VRT and LRT, ART is capable of removing a broader range of parauterine tissue. Patients may suffer from much more pain in the perioperative period due to wide resection and bleeding. Traditionally, ART has been deemed the standard gold treatment for early cervical cancer. Notably, since MIS (LRT and VRT) gradually gained popularity owing to perioperative advantages, several investigators have confirmed its safety and feasibility [5–9].

Of note, a multi-center randomized controlled trial (LACC trial) published in 2018 uncovered that patients receiving MIS were bound up with worse oncologic outcomes [10]. These results from the LACC trial have attracted paramount attention to the cancer outcomes of patients undergoing MIS, which is also necessary for us to conduct this meta-analysis. Thus our current work aims to evaluate the superiority between MIS and open surgery through fertility-sparing outcomes, oncologic outcomes, and perioperative pain, which might hopefully shed light on providing a precise and viable option for reproductive patients suffering from early-stage cervical cancer.

## Materials & methods

### Information selection

Information selection was carried on via four databases, including PubMed, Embase, Scopus, and Cochrane Library, for relevant studies and eligible articles published by three investigators on Aug 8, 2022, respectively. The Mesh terms include 'Uterine Cervical Neoplasms,' 'Trachelectomy,' 'Open Abdomen Techniques,' 'Abdomen,' 'Laparotomy,' 'Abdominal radical trachelectomy' and 'Minimally Invasive Surgical Procedures,' 'Laparoscopy.' The search formula that we used for advanced search in the PubMed database was as follows: ("laparotomy"[MeSH Terms] OR "Laparotomies"[Title/Abstract] OR "Minilaparotomy"[Title/Abstract] OR "Minilaparotomies"[Title/Abstract] OR "abdominal"[Title/Abstract] OR "Abdomen"[Title/Abstract] OR "Abdomens"[Title/Abstract] OR "open radical trachelectomy"[Title/Abstract]) AND ("laparoscopy"[MeSH Terms] OR "Laparoscopies"[Title/Abstract] OR "Celioscopy"[Title/Abstract] OR "Celioscopies"[Title/Abstract] OR "Peritoneoscopy"[Title/Abstract] OR "Peritoneoscopies"[Title/Abstract] OR "surgical procedures laparoscopic"[Title/Abstract] OR "laparoscopic surgical procedure"[Title/Abstract] OR "procedure laparoscopic surgical"[Title/Abstract] OR "procedures laparoscopic surgical"[Title/Abstract] OR "surgery laparoscopic"[Title/Abstract] OR "laparoscopic surgical procedures"[Title/Abstract] OR "laparoscopic surgery"[Title/Abstract] OR "laparoscopic surgeries"[Title/Abstract] OR "surgeries laparoscopic"[Title/Abstract] OR "laparoscopic assisted surgery"[Title/Abstract] OR "laparoscopic assisted surgeries"[Title/Abstract] OR (("Surgery"[MeSH Subheading] OR "Surgery"[All Fields] OR "surgical procedures, operative"[MeSH Terms] OR ("Surgical"[All Fields] AND "Procedures"[All Fields] AND "operative"[All Fields]) OR "operative surgical procedures"[All Fields] OR "general surgery"[MeSH Terms] OR ("general"[All Fields] AND "Surgery"[All Fields]) OR "general surgery"[All Fields] OR "surgery s"[All Fields] OR "surgerys"[All Fields] OR "Surgeries"[All Fields]) AND "laparoscopic assisted"[Title/Abstract]) OR "surgery laparoscopic assisted"[Title/Abstract] OR "surgical procedure laparoscopic"[Title/Abstract] OR "minimally invasive"[Title/Abstract]) AND ("trachelectomy"[MeSH Terms] OR "trachelectomy"[MeSH Terms] OR "trachelectomy"[MeSH Terms] OR "trachelectomy"[MeSH Terms] OR "trachelectomy"[MeSH Terms] OR "trachelectomy"[MeSH Terms] OR "trachelectomy"[MeSH Terms] OR "trachelectomy"[MeSH Terms] OR "trachelectomy"[MeSH Terms] OR "trachelectomy"[MeSH Terms] OR "trachelectomy"[MeSH Terms] OR "trachelectomy"[MeSH Terms]) AND ("uterine cervical neoplasms"[MeSH Terms] OR (("cervic"[All Fields] OR "cervicals"[All Fields] OR "cervices"[All Fields] OR "neck"[MeSH Terms] OR "neck"[All Fields] OR "Cervical"[All Fields] OR "uterine cervicitis"[MeSH Terms] OR ("Uterine"[All Fields] AND "cervicitis"[All Fields]) OR "uterine cervicitis"[All Fields] OR "cervicitis"[All Fields]) AND "neoplasm uterine"[Title/Abstract]) OR (("cervic"[All Fields] OR "cervicals"[All Fields] OR "cervices"[All Fields] OR "neck"[MeSH Terms] OR "neck"[All Fields] OR "Cervical"[All Fields] OR "uterine cervicitis"[MeSH Terms] OR ("Uterine"[All Fields] AND "cervicitis"[All Fields]) OR "uterine cervicitis"[All Fields] OR "cervicitis"[All Fields]) AND "neoplasms uterine"[Title/Abstract]) OR (("neoplasm s"[All Fields] OR "Neoplasms"[MeSH Terms] OR "Neoplasms"[All Fields] OR "Neoplasm"[All Fields]) AND "uterine cervical"[Title/Abstract]) OR "neoplasms uterine cervical"[Title/Abstract] OR "uterine cervical neoplasm"[Title/Abstract] OR "neoplasms cervical"[Title/Abstract] OR "cervical neoplasms"[Title/Abstract] OR "cervical neoplasm"[Title/Abstract] OR "neoplasm cervical"[Title/Abstract] OR "neoplasms cervix"[Title/Abstract] OR "cervix neoplasms"[Title/Abstract] OR "cervix neoplasm"[Title/Abstract] OR (("neoplasm s"[All Fields] OR "Neoplasms"[MeSH Terms] OR "Neoplasms"[All Fields] OR "Neoplasm"[All Fields]) AND "Cervix"[Title/Abstract]) OR "cancer of the uterine cervix"[Title/Abstract] OR "cancer of the cervix"[Title/Abstract] OR "cervical cancer"[Title/Abstract] OR "uterine cervical cancer"[Title/Abstract] OR "cancer uterine cervical"[Title/Abstract] OR "cancers uterine cervical"[Title/Abstract] OR "cervical cancer uterine"[Title/Abstract] OR "cervical cancers uterine"[Title/Abstract] OR "uterine cervical cancers"[Title/Abstract] OR "cancer of cervix"[Title/Abstract] OR "cervix cancer"[Title/Abstract] OR "cancer cervix"[Title/Abstract] OR "cancers cervix"[Title/Abstract]).

### Inclusion and exclusion criteria

The inclusion criteria including:

1. at reproductive age (< 49 years old);
2. clinical stage IA1 to IB2;
3. received RT surgery;
4. cervical cancer diagnoses were confirmed;

The exclusion criteria including:

1. age ≧ 49 years;
2. previous subtotal hysterectomy history;
3. patients with a history of invasive injury that may lead to severe adhesions during surgery.

### Data collection

Two authors carried out the data collection procedure, respectively. Microsoft Excel 2019 was utilized to collect the designated and summarize the alternative data. The following data and information were extracted:

1. Basic information: Age, FIGO stage, BMI, surgical time, size of the tumor, length of the tumor, depth of tumor, follow-up time, histology grade, presence of LVSI, race.
2. Fertility-sparing outcomes: clinical pregnancy rate, pregnancy miscarriage rate, pregnancy rate (Third-trimester deliveries Pre-term rate), Pregnancy rate (Third-trimester deliveries term rate).
3. Cancer outcomes: the overall survival, recurrence rate.
4. Surgery-related outcomes: estimated blood loss (ml), blood transfusion, postoperative complications.

If dissenting opinions occur during the quality assessment between the two investigators, the disputed study or data would be sent to the third-party investigator to decide the final results.

### Quality assessment

Two researchers evaluated the quality of the RCTs following the Cochrane handbook. The Cochrane handbook concentrates on assessing the risk of bias, and selection bias, performance bias, detection bias, attrition bias, and reporting bias are included, with the level of low risk, unclear risk, and high risk on each item of bias assessment. Cochrane risk of bias tool was utilized with Review Manager V.5.4.

The Jadad scale was conducted to evaluate the quality of each trial, with seven items including the description of randomization mentioned, appropriate randomization method, randomization concealment, appropriate concealment of randomization method, blinding, appropriate blinding method and reporting of withdrawals, accounting for 1 in each item. Ultimately, studies that gained 4 points or over 4 points would be deemed high-quality clinical trials.

In accordance with the GRADE Handbook, we have assessed the quality of the outcomes on the basis of five downgrading factors (risk of bias, inconsistency, indirectness, imprecision, and publication bias) and three upgrading factors (large effect, plausible residual ronfounding, dose–response gradient). GRADE profile software was used to produce a summary of findings tables.

If dissenting opinions occur during the quality assessment between the two investigators, the disputed study or data would be sent to the third-party investigator to decide the final results.

### Data synthesis

In this process, we analyzed data from selected studies using Review Manager V.5.4. The odds ratios (OR) and 95% confidence interval (CI) were calculated and visualized with the forest plot in Review Manager V.5.4. Secondly, we accessed the mean value and SD from continuous results. Ultimately, we measured these outcomes by using a random-effects model. The heterogeneity of every statistical test could be seen from the I^2^ value. We need to consider the following explanations: 0–40% implied low heterogeneity; 50–70% exhibited medium heterogeneity, while > 70% means extremely high heterogeneity.

In addition, to reduce the heterogeneity of primary outcomes, it is necessary to conduct a sensitivity analysis by excluding literature, and subgroup analysis is conducted when clinical characteristics are complete in every included study. Moreover, Egger's test was applied to evaluate the publication bias of each primary outcome. The two-tailed *P*-value < 0.05 was deemed statistical significance, which indicated a positive result in the primary outcome. Besides, this meta-analysis abides by the PRISMA guidelines and the AMSTAR checklist for meta-analysis and systematic review.

## Results

### Study selection

**Fig. 1:**
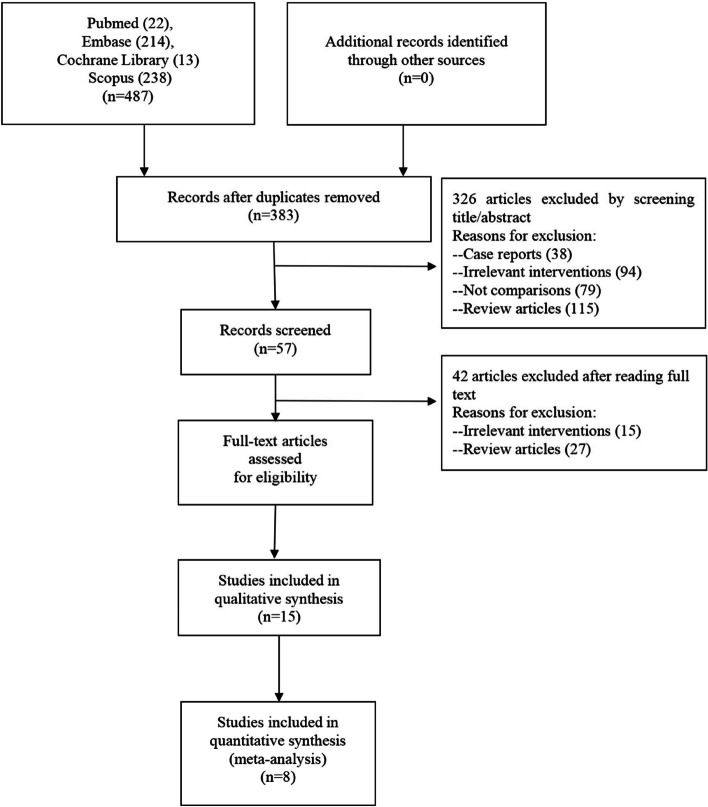
Selection flowchart of included studies

Four hundred eighty-seven studies were selected from PubMed (*n* = 22), Embase (*n* = 214), Cochrane Library (*n* = 13), and Scopus (*n* = 238). We retained three hundred and eighty-three references after removing duplicate allusions. Through the primary inspecting of titles and abstracts, we took out 326 articles, including case reports (*n* = 38), irrelevant interventions (*n* = 94), no comparisons (*n* = 79), and review articles (*n* = 115). After reserving 57 articles, we complementary removed 42 articles, consisting of irrelevant interventions (*n* = 15) and review articles (*n* = 27). Eventually, eight studies were retained, and methods for each study have previously been published [5, 11–17] (Fig. 1).

### Study characteristics

In the eight clinical trials from June 2002 to December 2017, 1361 early-stage cervical cancer patients were assigned to the open invasive radical trachelectomy group (*n* = 706) or the minimally invasive radical trachelectomy group (*n* = 655). Firstly, MIS procedures included LRT, RRT, and VRT. Secondly, the mean age varied from 27.33 to 36.88 years, and the mean BMI varied from 22.01 kg/m2 to 25.06 kg/m2. Moreover, the mean follow-up time ranged from 9 months to 113.56 months. Furthermore, the number of patients in the five trials in FIGO stage IA1 ranged from 0 to 27, the number of patients in the seven trials in FIGO stage IA2 ranged from 0 to 51, the number of patients in the seven trials in FIGO stage IB1 ranged from 8 to 307. Not only that, the number of SCC patients in the five trials ranged from 5 to 234, the number of Adenocarcinoma patients in the six trials ranged from 0 to 115, the number of patients with other histology in the three trials ranged from 2 to 16.

The main characteristic of each study is summarized in Tables 1 and 2.

**Table 1:** Primary characteristics of included studies

Trials	Year	Country	Time Range	Minimally Invasive Surgery	Sample Size			Mean Age (year)		BMI		Follow-up Time (month)	
					Total	Open	MIS	Open	MIS	Open	MIS	Open	MIS
He	2022	Asia-China	January 2005 to June 2017	LRT	33	18	15	30.00 ± 4.30		---	---	113.56 ± 46.12	28.00 ± 15.45
Kucukmetin	2014	Europe-United Kingdom	2004 to 2013	LRT	27	16	11	27.33 ± 3.39	29.79 ± 4.71	24.5 ± 2.54	23.20 ± 4.1	43 ± 25.5	9 ± 4.75
Matsuo	2019	North America-America	2010 to 2015	LRT or RRT	246	102	144	30.41 ± 6.02	31.70 ± 5.99	---	---	44.57 ± 30.83	37 ± 20.97
Rodolakis	2018	Europe-Athens	---	LRT or VRT	28	13	15	---	---	---	---	---	---
Salvo	2022	North America-USA	January 2005 to December 2017	LRT or RRT	646	358	288	31.95 ± 3.77	31.03 ± 4.73	22.01 ± 3.5	23.97 ± 5.7	68.94 ± 43.77	31.40 ± 42.72
Shen	2013	Asia-China	---	VRT	145	73	72	---	---	---	---	23.4 ± 26.8	32.2 ± 26.8
Vieira	2015	North America-USA	June 2002 to July 2013	LRT or RRT	100	58	42	29.52 ± 4.20	30.67 ± 3.49	23.85 ± 3.4	25.06 ± 6.0	68.08 ± 29.57	27.83 ± 13.56
Wang	2021	Asia-China	---	VRT	136	68	68	36.88 ± 9.93	35.14 ± 10.31	23.17 ± 3.4	22.79 ± 3.1	---	---

**Table 2:** Cancer characteristics of included studies

Trials	Year	FIGO Stage IA1		FIGO Stage IA2		FIGO Stage IB1		SCC		Adenocarcinoma		Other Histology	
		Open	MIS	Open	MIS	Open	MIS	Open	MIS	Open	MIS	Open	MIS
He	2022	3	2	2	0	13	13	---	---	---	---	---	---
Kucukmetin	2014	0	0	0	0	11	16	13	5	3	6	---	---
Matsuo	2019	17	27	12	13	73	107	51	60	40	68	---	---
Rodolakis	2018	---	---	0	3	12	10	---	---	0	6	---	---
Salvo	2022	---	---	51	46	307	242	234	168	108	115	16	5
Shen	2013	---	---	---	---	---	---	---	---	---	---	---	---
Vieira	2015	3	3	13	12	42	27	29	20	22	20	7	2
Wang	2021	6	4	22	16	9	8	45	49	14	11	9	8

### Quality assessment

Following the Cochrane handbook, firstly, in terms of the random sequence generation in selection bias, two trials were concerned with low risk, three articles were unclear, and three articles were concerned with high risk; In terms of the allocation concealment in selection bias, only one trial was concerned as low risk, and one article was concerned with high risk, but six articles were unclear. Secondly, four trials were at an unclear bias in the risk of performance bias, and four articles were concerned with high risk. Moreover, seven articles were estimated as low risk in the detection bias, and only one study was at unclear risk. Ultimately, attrition bias was at low risk in all trials. Regarding reporting bias, seven trials were considered low-risk, and only one article was unclear (Figure S1).

Simultaneously, each item on the Jadad scale scored between 1 and 7, and trials with four or more were considered high-quality trials. In our meta-analysis, five trials scored four points or more, assessed as high-quality, while three studies were under four points (Table S1).

Among the 8 outcomes analyzed via the GRADE approach in this meta-analysis, except for the estimated blood loss, which was of low quality, the other seven, including the pregnancy rate, were of very low quality. The majority of the studies had a short follow-up time and incomplete follow-up, and the retrospective character of the included research will increase the risk of bias. A portion of the study seems to show that CI overestimation resulted in accuracy degradation. Future research should use a more rigorous design and sufficient statistical analysis (Table S2).

### Primary outcome

#### Analysis of fertility-sparing outcomes

##### Pregnancy rate (Third-trimester delivery)

Three studies of the Pregnancies-Third trimester deliveries included 224 patients. The pooled analysis showed that there was an extremely significant difference in pregnancies-Third trimester deliveries between the Open group and MIS group [OR = 2.68; 95% CI (1.29, 5.59); P = 0.008], with high heterogeneity (I^2^ = 29%). Compared with alternative studies, the research by Rodolakis revealed in 2014 manifested prominent heterogeneity. After deleting this study, heterogeneity was low (I^2^ = 0%). Not only that, the comprehensive effects revealed that the Pregnancies-Third trimester deliveries to a highly significant difference in both groups [OR = 3.90; 95% CI (1.66, 9.18); *P* = 0.002]. In the research by Rodolakis, uterine artery preservation is described in 4 cases of ART during pregnancy, while other studies did not record uterine artery preservation. The Egger's test assessed the publication bias of Pregnancies-Third trimester deliveries, which showed no publication bias.

##### Analysis of pregnancy (Second-trimester delivery)

Three studies reporting the results of pregnancies-Second trimester deliveries included 148 patients. The pooled analysis showed that there was no difference in pregnancies-Second trimester deliveries [OR = 1.54; 95% CI (0.42, 5.65); *P* = 0.52] between the Open group and the MIS group, with low heterogeneity(I^2^ = 0%). The Egger's test assessed the publication bias of pregnancies-Second trimester deliveries, which showed no publication bias.

##### Analysis of pregnancy miscarriage rate

Three studies reporting the results of pregnancy miscarriage included 237 patients. The pooled analysis showed that there was no difference in pregnancy miscarriage [OR = 1.94; 95% CI (0.61, 6.21); *P* = 0.26] between the Open group and the MIS group, with low heterogeneity(I^2^ = 0%). Egger's test assessed the publication bias of pregnancy miscarriage, which showed no publication bias.

##### Analysis of clinical pregnancy rate

Four studies reporting the results of clinical pregnancy rate included 303 patients. There was a significant distinction in clinical pregnancy [OR = 0.70; 95% CI (0.24, 0.71); *P* = 0.001] between the Open group and the MIS group, with high heterogeneity(I^2^ = 69%). Compared with alternative studies, the research by Wang revealed in 2021, and the research by Shen revealed in 2013 manifested prominent heterogeneity. After deleting the above study, heterogeneity was low (I^2^ = 0%). However, there was no significant distinction in clinical pregnancy [OR = 2.39; 95% CI (0.61, 9.39); *P* = 0.21]. In the research by Shen and Wang, patients were conducted with radical vaginal trachelectomy and laparoscopic-assisted vaginal radical trachelectomy, respectively, while other studies were laparoscopic radical trachelectomy. The publication bias of the clinical pregnancy rate was assessed in Egger's test, and no publication bias was detected.

#### Analysis of cancer outcomes

##### Analysis of overall survival

Five studies reporting the results of the overall survival included 1170 patients. The pooled analysis showed that there was no difference in the overall survival [OR = 1.56; 95% CI (0.70, 3.45); *P* = 0.27] between the Open group and the MIS group, with low heterogeneity(I^2^ = 0%). The publication bias of the overall survival was assessed in Egger's test, which showed no publication bias.

##### Analysis of recurrence rate

Five studies reporting the results of recurrence included 951 patients. The pooled analysis showed that there was no difference in recurrence [OR = 0.63; 95% CI (0.35, 1.12); *P* = 0.12] between the Open group and the MIS group, with low heterogeneity(I^2^ = 0%). The publication bias of recurrence was assessed in Egger's test, which showed no publication bias.

#### Analysis of surgery-related outcomes

##### Analysis of the estimated blood loss

Five studies reporting the results of estimated blood loss included 809 patients. The pooled analysis showed that there was an extremely significant difference in estimated blood loss between the Open group and MIS group [MD = 227.92; 95% CI (186.51, 269.19); *P* < 0.00001], with high heterogeneity (I^2^ = 81%). Compared with alternative studies, the research by He revealed in 2022, and the research by Vieira revealed in 2015 manifested prominent heterogeneity. After deleting the above two studies, heterogeneity was low (I^2^ = 0%). Not only that, the comprehensive effects revealed that the estimated blood loss to a highly significant difference in both groups [MD = 139.40; 95% CI (79.05, 199.75); *P* < 0.0001]. In the research by He, 72.73% of the patients were diagnosed with SCC (squamous cell carcinoma, SCC), while the ratio of SCC was lower than that in other studies. Meanwhile, in Vieira's study, the number of patients at the 1BI FIGO stage significantly differed between the Open group and the MIS group. Egger's test assessed the publication bias of estimated blood loss, which showed no publication bias.

##### Analysis of the postoperative complication rate

Three studies reporting the results of recurrence included 263 patients. The pooled analysis showed that there was no difference in postoperative complications between the Open group and the MIS group [OR = 1.52; 95% CI (0.89, 2.60); *P* = 0.12], with low heterogeneity(I^2^ = 0%). The publication bias of postoperative complications was assessed in Egger's test, which showed no publication bias.

## Discussion

At present, a growing number of studies have shown that minimally invasive radical trachelectomy is safe and feasible. Many researchers have sought to evaluate the effect of open and minimally invasive radical trachelectomy for cervical resection on fertility preservation and cancer outcomes in patients with early-stage cervical cancer. Notably, a 2018 multi-center prospective randomized trial (LACC) trial found that minimally invasive radical hysterectomy related to lower disease-free survival, overall survival rates, and higher recurrence rates [10]. To validate the superiority between open surgery and minimally invasive surgery, we conducted a meta-analysis to assess the fertility-sparing outcomes, including (1) pregnancy rate (Third-trimester delivery). (2) pregnancy rate (Second-trimester delivery). (3) miscarriage rate. (4) clinical pregnancy rate; and cancer outcomes, including (1) overall survival. (2) recurrence rate. This study aims to provide a reference for patients suffering from early-stage cervical cancer to preserve their fertility with a more appropriate resection procedure.

In our study, 1361 patients were included in eight studies. Five of these were high-quality assessed by the Jadad scale, while three studies were of low quality via assessment of the Jadad scale.

**Fig. 2:**
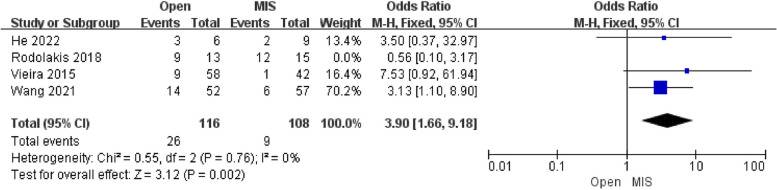
Forest plot of pregnancy rate (Third-trimester delivery)

**Fig. 3:**
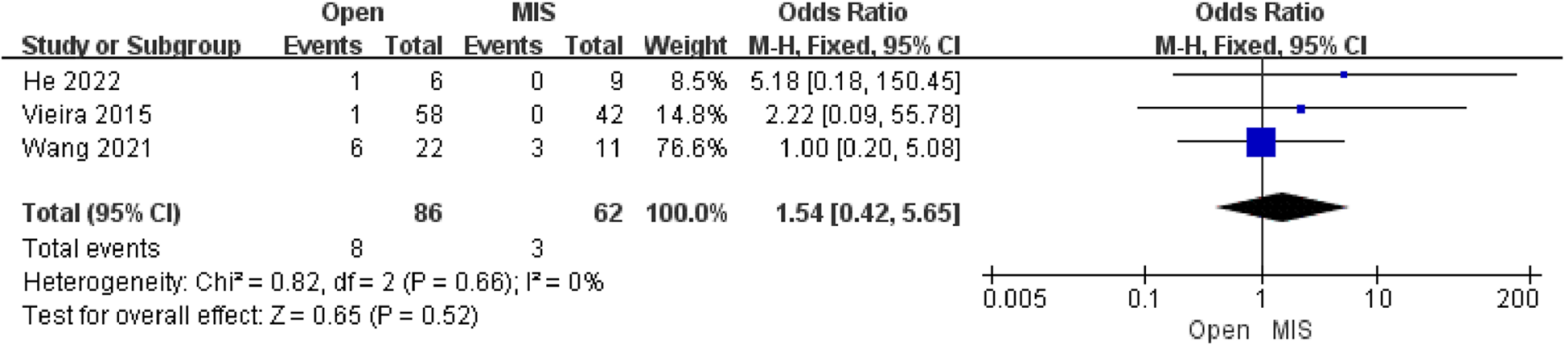
Forest plot of pregnancy (Second-trimester delivery)

Concerning the pregnancy rate (Third-trimester delivery) shown in Fig. 2, our pooled analysis found that the pregnancy rate of third-trimester delivery in the Open group is significantly higher than in the MIS group. Notably, the Open group has a slightly higher rate of pregnant rate of second-trimester delivery though no statistical difference was found in Fig. 3. On the one hand, patients with ART would not choose pregnancy for months until the uterus is viable [18], which was bound up with the higher rate of second and third-trimester delivery. On the other hand, ART was introduced early, and the technology of ART was maturer and more standardized, increasing the pregnant rate of second and third-trimester delivery [5].

**Fig. 4:**
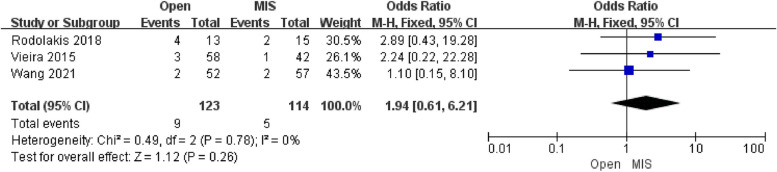
Forest plot of pregnancy miscarriage rate

**Fig. 5:**
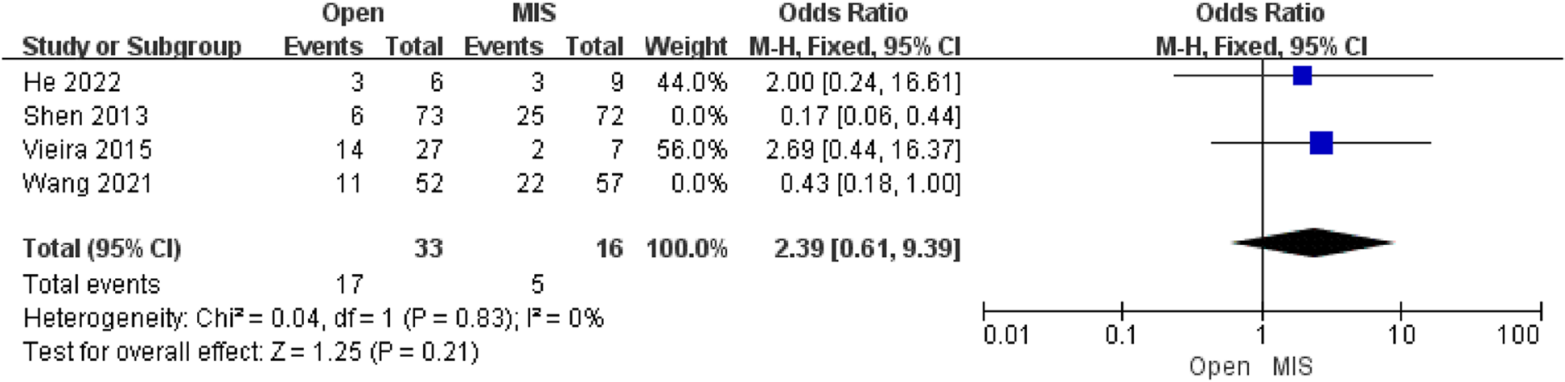
Forest plot of clinical pregnancy rate

Moreover, the clinical pregnancy rate and pregnancy miscarriage rate did not differ between the Open group and MIS group in Figs. 4 and 5, respectively. Notably, it can be referred to Figs. 4 and 5 that the Open group had a slightly lower clinical pregnancy rate and higher miscarriage rate. The reason may be a cervical factor that the residual cervix of patients undergoing ART is shorter than that of patients undergoing minimally invasive surgery, so patients with ART may secrete less cervical mucus and more easily be exposed to the risk of premature rupture of membranes after pregnancy [17].

**Fig. 6:**
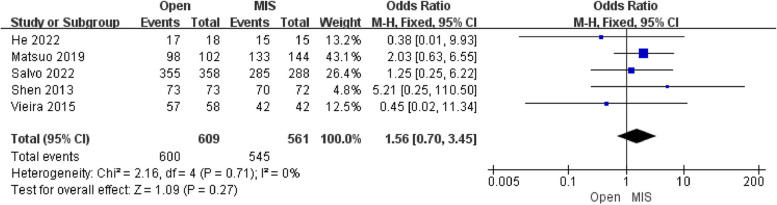
Forest plot of overall survival

**Fig. 7:**
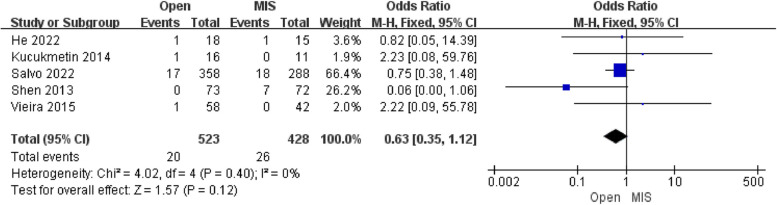
Forest plot of recurrence rate

Simultaneously, no significant difference was found in the overall survival and recurrence between the Open group and the MIS group in contrast to previous studies demonstrating inferior survival for minimally invasive compared with the Open group, which provided grounds for discussion and counseling patients with early cervical cancer who wish to preserve future fertility (Figs. 6, 7). Due to poor cases in this study, the majority of patients were on IB1 FIGO stage, potentially related to a subjective result of recurrence rate and overall survival [19]. In terms of the risk factors of recurrence rate and overall survival, the previous combined case series have shown in the following lines: (1) Insufficient parametrial excision [20]. (2) Lesion size > 2 cm. [21] (3) Lymphovascular space involvement [19]. Besides, there is controversy as to whether adenocarcinoma or adenosquamous histology is associated with a higher risk of recurrence compared to squamous cell carcinomas of the cervix [22].

**Fig. 8:**
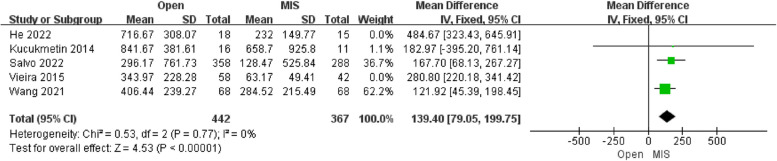
Forest plot of the estimated blood loss

In this study, the estimated blood loss in the MIS group was less than that in the open group, which was shown in Fig. 8, consistent with the report results in most literature. As Einstein et al. [23] compared the scope of resection between 28 cases of VRT and 15 cases of ART and found that the average width of parauterine tissue resection was 1.45 cm in VRT and 3.97 cm in ART, demonstrating a statistically significant difference. Previously because of this, ART has broader indications than MIS but with worse blood loss.

**Fig. 9:**
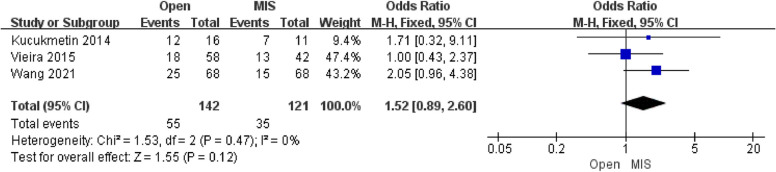
Forest plot of the postoperative complication rate

There is no statistically significant difference in postoperative complication rate between the two groups, and no cervical stenosis, external iliac vein injury, and rectal dysfunction occurred, contrary to the multiple phase III randomized trials [24], which reported decreased postoperative complication rates with MIS hysterectomy compared to the Open Group (Fig. 9) [25].

As evinced by previous systematic reviews, this study has reflected different results. In terms of fertility-sparing outcomes, Bentivegna [26] and Smith [27] suggested that the pregnancy rate was higher in patients submitted to MIS compared with ART, which was opposite to Nezhat’s study and this study [28]. In addition, Bentivegna et al. found that the pregnancy rate is significantly higher in patients undergoing ART. However, this study has no significant difference in the pregnancy rate. Moreover, our study, likewise Nezhat's study, demonstrated that there was no difference in second-trimester delivery in different surgery. Ultimately, when it comes to overall survival and recurrence, this study reported no difference in recurrence between the Open group and the MIS group, which was consistent with Nezhat’s study. When considering the previous review, several limitations should be aware. Firstly, data are not being directly compared in statistical analysis, making it difficult to discern whether the determined values for one group are within or outside the margin of error for another group. Besides, the previous reviews lacked quality assessment of included studies, which could not probably avoid data bias to a certain extent in previous studies.

It is worth noting that the results from LACC trial implied that patients received open surgery might have a better prognosis, which led to the declination of the number of patients treated with MIS in Italy. A multi-center retrospective study in Italy manifested the declination of MIS surgery did not alter the post-operative complication rate. By occasion of limited follow-up time, this retrospective study is under paucity of the information in prognosis data [29]. Simultaneously, the other landmark clinical trial, CX.5/SHAPE trial conducted in Canada corroborated there existed possibly non-inferiority between radical and simple hysterectomy (including MIS). Substantially, due to insufficient evidence concerning fertility-sparing oucomes and cancer-related outcomes from different countries and areas after LACC trial and SHAPE trial, we still hold a prudent and conservative attitude towards the reduction of MIS and radical surgery in clinical practice.

There were several limitations of our meta-analysis. In the first place, the sample size was small (1369 patients). Secondly, there was heterogeneity in the follow-up period, the preservation of uterine arteries, and the histology situation in each study. Meanwhile, with the increasing trend in minimally invasive surgery, comparisons have often been flawed by a sequential pattern, and cases could not be concurrently evaluated. Last but not least, although there is no instinct difference between the overall survival and recurrence according to the included studies, the tumor stage and intraoperative lymph node dissection should still be considered. Further RCTs should be conducted to provide stronger and more objective evidence of the superiority between Open and MIS.

## Conclusion

Our pooled analysis suggested that patients in the open group were more likely to reach third-trimester pregnancy delivery than the MIS group. At the same time, the MIS group had fewer estimated blood loss. Simultaneously, our study found that there was no overt difference in the occurrence of events in second-trimester pregnancy, miscarriage rate, clinical pregnancy rate, overall survival, recurrence and postoperative complications between the Open group and MIS group. Finally, the above results summarize that the open group may have more advantages in fertility preservation, maybe a better therapeutic option. Of note, due to insufficient cases in this study, a more robust conclusion requires more relevant articles in the future.

### Supplementary Information

**Additional file 1.**

**Additional file 2.**

**Additional file 3.**

## Data Availability

The datasets used and analyzed during the current study are available from the corresponding author upon reasonable request.

## Abbreviations

RT: Radical trachelectomy
ART: Abdominal radical trachelectomy
LRT: Laparoscopic radical trachelectomy
RRT: Robot-assisted radical trachelectomy
MIS: Minimally invasive surgery
LVSI: Lymphatic vascular invasion
AC: Adenocarcinoma
SCC: Squamous cell carcinoma
BMI: Body mass index
PROM: Prelabour rupture of the membranes

## References

[1] Bays HE, Jones PH, Orringer CE, Brown WV, Jacobson TA (2016). National lipid association annual summary of clinical lipidology 2016. J Clin Lipidol.

[2] Jacobson TA, Ito MK, Maki KC, Orringer CE, Bays HE, Jones PH (2015). National lipid association recommendations for patient-centered management of dyslipidemia: part 1–full report. J Clin Lipidol.

[3] Siegel RL, Miller KD, Jemal A (2020). Cancer statistics, 2020. CA Cancer J Clin.

[4] Zhang XH, Chen YM, Sun Y, Qiu LQ (2020). Analysis on the birth situation different fertility policy periods in monitoring area of birth defect population in Zhejiang Province. Zhonghua Yu Fang Yi Xue Za Zhi.

[5] Kucukmetin A, Biliatis I, Ratnavelu N, Patel A, Cameron I, Ralte A (2014). Laparoscopic radical trachelectomy is an alternative to laparotomy with improved perioperative outcomes in patients with early-stage cervical cancer. Int J Gynecol Cancer.

[6] Lu Q, Liu C, Zhang Z (2014). Total laparoscopic radical trachelectomy in the treatment of early-stage cervical cancer: review of technique and outcomes. Curr Opin Obstet Gynecol.

[7] Park NY, Chong GO, Cho YL, Park IS, Lee YS (2009). Total laparoscopic nerve-sparing radical trachelectomy. J Laparoendosc Adv Surg Tech A.

[8] Ramirez PT, Schmeler KM, Malpica A, Soliman PT (2010). Safety and feasibility of robotic radical trachelectomy in patients with early-stage cervical cancer. Gynecol Oncol.

[9] Vieira MA, Rendón GJ, Munsell M, Echeverri L, Frumovitz M, Schmeler KM (2015). Radical trachelectomy in early-stage cervical cancer: a comparison of laparotomy and minimally invasive surgery. Gynecol Oncol.

[10] Ramirez PT, Frumovitz M, Pareja R, Lopez A, Vieira M, Ribeiro R (2018). Minimally invasive versus abdominal radical hysterectomy for cervical cancer. N Engl J Med.

[11] Cao DY, Yang JX, Wu XH, Chen YL, Li L, Liu KJ (2013). Comparisons of vaginal and abdominal radical trachelectomy for early-stage cervical cancer: preliminary results of a multi-center research in China. Br J Cancer.

[12] He Z, Bian C, Xie C (2022). Fertility-sparing surgery in early-stage cervical cancer: laparoscopic versus abdominal radical trachelectomy. BMC Womens Health.

[13] Matsuo K, Chen L, Mandelbaum RS, Melamed A, Roman LD, Wright JD (2019). Trachelectomy for reproductive-aged women with early-stage cervical cancer: minimally invasive surgery versus laparotomy. Am J Obstet Gynecol.

[14] Rodolakis A, Thomakos N, Sotiropoulou M, Kypriotis K, Valsamidis D, Bourgioti C (2018). Abdominal radical trachelectomy for early-stage cervical cancer during pregnancy: a provocative surgical approach. Overview of the literature and a single-institute experience. Int J Gynecol Cancer.

[15] Salvo G, Ramirez PT, Leitao M, Cibula D, Fotopoulou C, Kucukmetin A (2019). International radical trachelectomy assessment: IRTA study. Int J Gynecol Cancer.

[16] Salvo G, Ramirez PT, Leitao MM, Cibula D, Wu X, Falconer H (2022). Open vs minimally invasive radical trachelectomy in early-stage cervical cancer: international radical trachelectomy assessment study. Am J Obstet Gynecol..

[17] Wang Y, Wang A, Zhan J, Guo T (2021). Curative effect of laparoscopic-assisted vaginal radical trachelectomy combined with pelvic lymph node dissection on early-stage cervical cancer. J buon.

[18] Ungár L, Smith JR, Pálfalvi L, Del Priore G (2006). Abdominal radical trachelectomy during pregnancy to preserve pregnancy and fertility. Obstet Gynecol.

[19] Delgado G, Bundy B, Zaino R, Sevin BU, Creasman WT, Major F (1990). Prospective surgical-pathological study of disease-free interval in patients with stage IB squamous cell carcinoma of the cervix: a Gynecologic Oncology Group study. Gynecol Oncol.

[20] Kim SI, Cho JH, Seol A, Kim YI, Lee M, Kim HS (2019). Comparison of survival outcomes between minimally invasive surgery and conventional open surgery for radical hysterectomy as primary treatment in patients with stage IB1-IIA2 cervical cancer. Gynecol Oncol.

[21] Plante M, Renaud MC, François H, Roy M (2004). Vaginal radical trachelectomy: an oncologically safe fertility-preserving surgery. An updated series of 72 cases and review of the literature. Gynecol Oncol.

[22] Marchiole P, Benchaib M, Buenerd A, Lazlo E, Dargent D, Mathevet P. Oncological safety of laparoscopic-assisted vaginal radical trachelectomy (LARVT or Dargent’s operation): a comparative study with laparoscopic-assisted vaginal radical hysterectomy (LARVH). Gynecol Oncol. 2007;106(1):132–41. 10.1016/j.ygyno.2007.03.009 17493666

[23] Einstein MH, Park KJ, Sonoda Y, Carter J, Chi DS, Barakat RR (2009). Radical vaginal versus abdominal trachelectomy for stage IB1 cervical cancer: a comparison of surgical and pathologic outcomes. Gynecol Oncol.

[24] Janda M, Gebski V, Davies LC, Forder P, Brand A, Hogg R (2017). Effect of total laparoscopic hysterectomy vs total abdominal hysterectomy on disease-free survival among women with stage i endometrial cancer: a randomized clinical trial. JAMA.

[25] Walker JL, Piedmonte MR, Spirtos NM, Eisenkop SM, Schlaerth JB, Mannel RS (2009). Laparoscopy compared with laparotomy for comprehensive surgical staging of uterine cancer: gynecologic oncology group study LAP2. J Clin Oncol.

[26] Bentivegna E, Maulard A, Pautier P, Chargari C, Gouy S, Morice P (2016). Fertility results and pregnancy outcomes after conservative treatment of cervical cancer: a systematic review of the literature. Fertil Steril.

[27] Smith ES, Moon AS, O’Hanlon R, Leitao MM Jr, Sonoda Y, Abu-Rustum NR, et al. Radical trachelectomy for the treatment of early-stage cervical cancer: a systematic review. Obstet Gynecol. 2020;136(3):533–42. 10.1097/AOG.0000000000003952 PMC7528402 32769648

[28] Nezhat C, Roman RA, Rambhatla A, Nezhat F (2020). Reproductive and oncologic outcomes after fertility-sparing surgery for early stage cervical cancer: a systematic review. Fertil Steril.

[29] Bogani G, Donato VD, Scambia G, Landoni F, Ghezzi F, Muzii L (2022). Practice patterns and 90-day treatment-related morbidity in early-stage cervical cancer. Gynecol Oncol.

