# Supplementary figures and images for: A meta-analysis of treatment for early-stage cervical cancer: open versus minimally invasive radical trachelectomy
Source: BMC Pregnancy Childbirth. 2023 Oct 14;23:727. doi: 10.1186/s12884-023-06036-z (PMC10576315; doi:10.1186/s12884-023-06036-z)

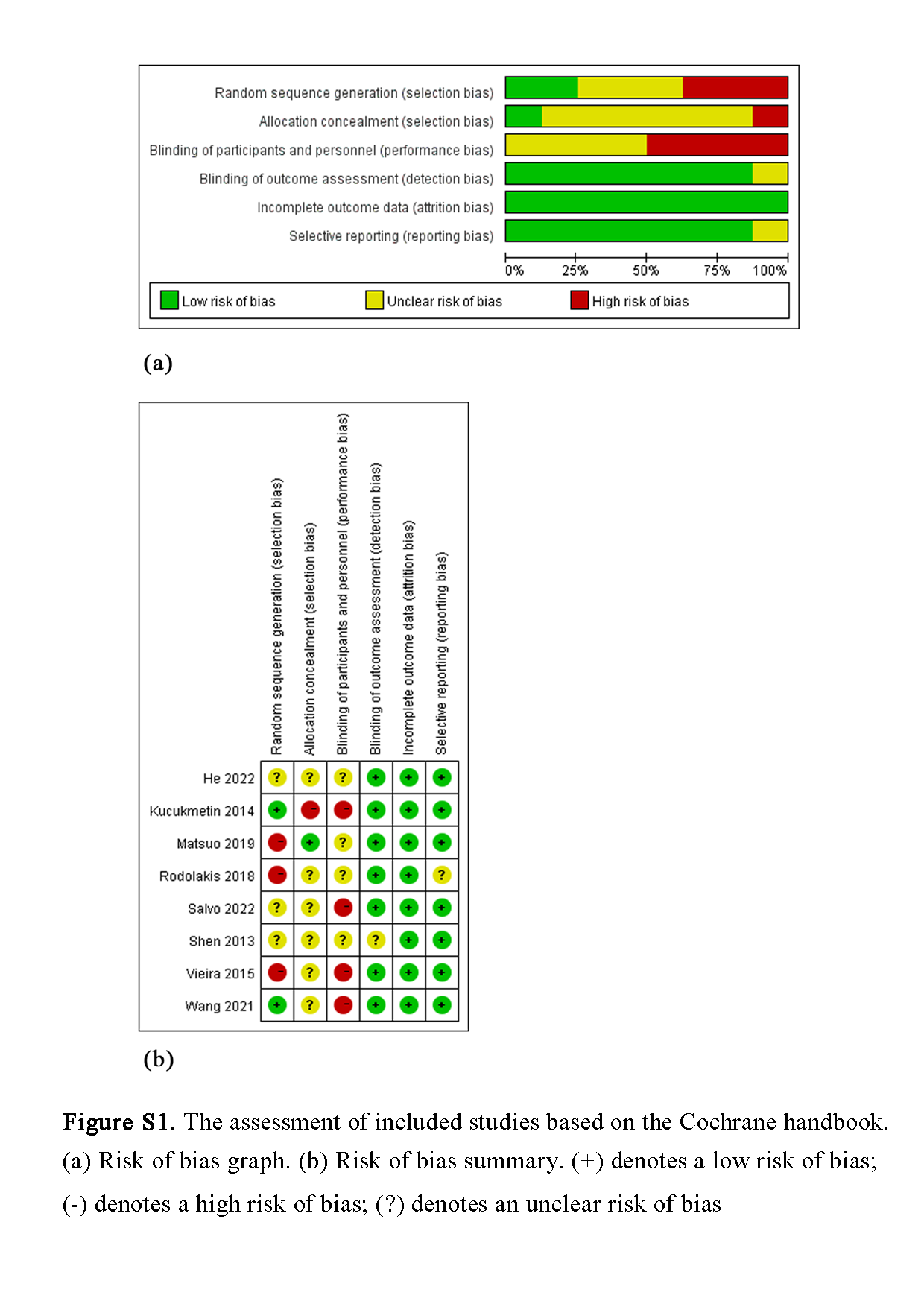

Supplement: Supplementary file 1 — Additional file 1. [file 12884_2023_6036_MOESM1_ESM.png]

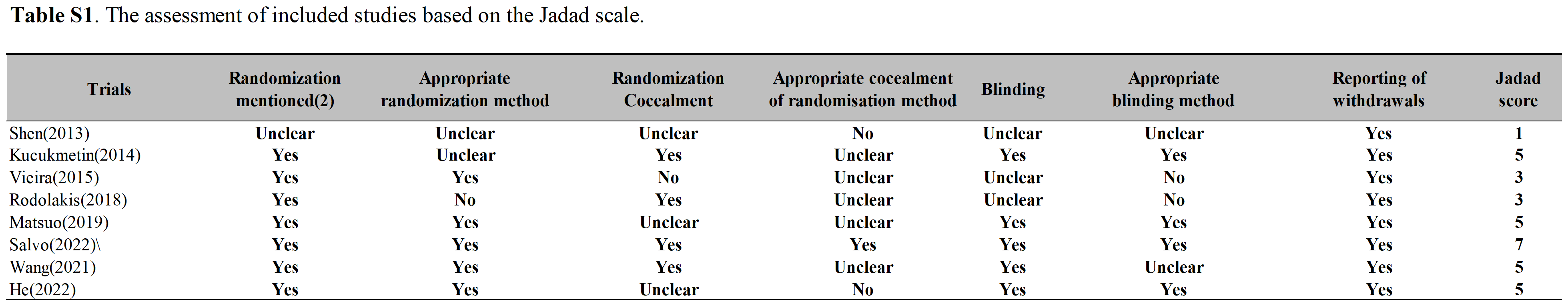

Supplement: Supplementary file 2 — Additional file 2. [file 12884_2023_6036_MOESM2_ESM.png]

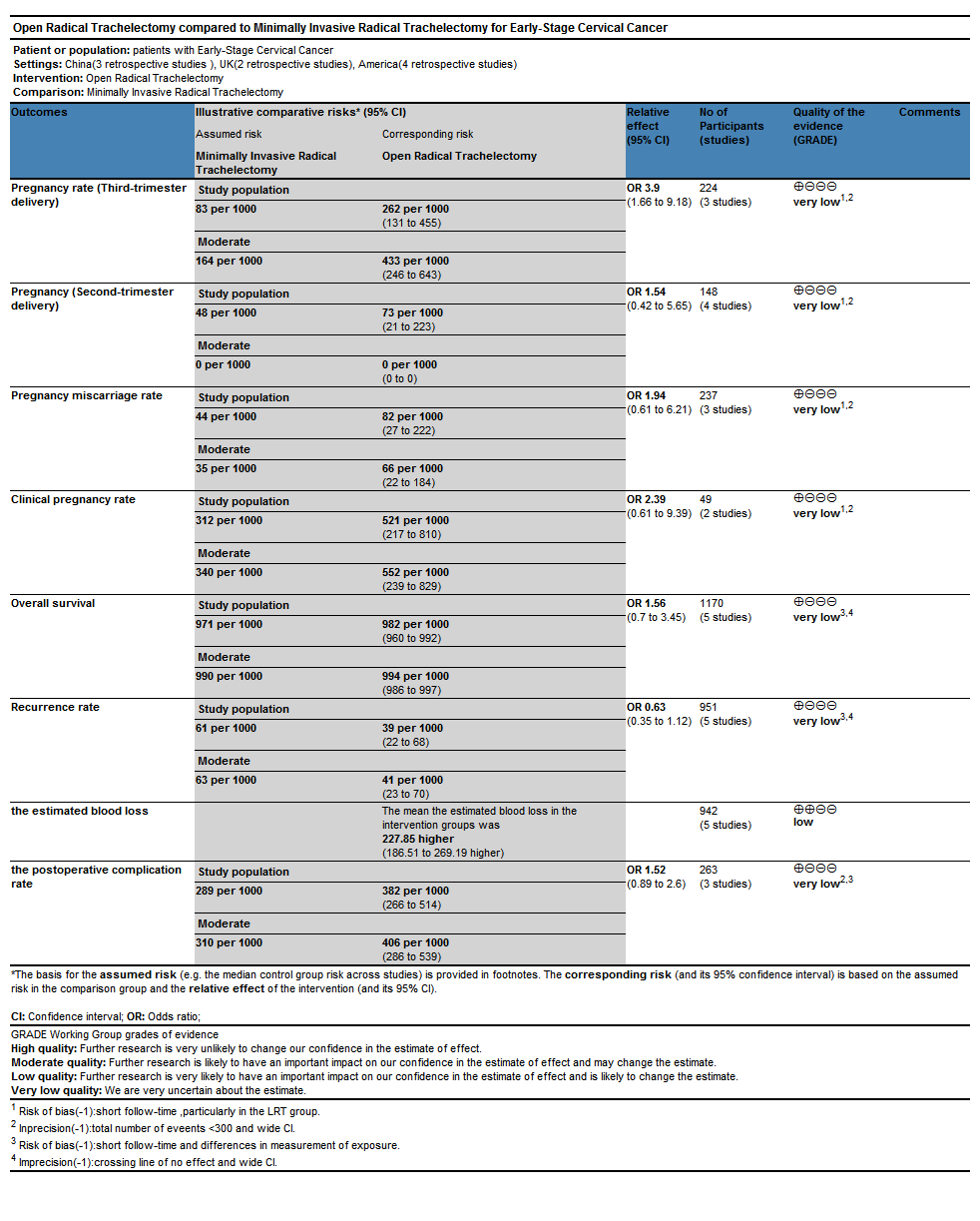

Supplement: Supplementary file 3 — Additional file 3. [file 12884_2023_6036_MOESM3_ESM.png]
